# Novel Tn*4371*-ICE like element in *Ralstonia pickettii *and Genome mining for comparative elements

**DOI:** 10.1186/1471-2180-9-242

**Published:** 2009-11-26

**Authors:** Michael P Ryan, J Tony Pembroke, Catherine C Adley

**Affiliations:** 1 Microbiology Laboratory, Department of Chemical and Environmental Sciences, University of Limerick, Limerick, Ireland; 2 Molecular Biochemistry Laboratory, Department of Chemical and Environmental Sciences, University of Limerick, Limerick, Ireland

## Abstract

**Background:**

Integrative Conjugative Elements (ICEs) are important factors in the plasticity of microbial genomes. An element related to the ICE Tn*4371 *was discovered during a bioinformatic search of the *Ralstonia pickettii *12J genome. This element was analysed and further searches carried out for additional elements.

A PCR method was designed to detect and characterise new elements of this type based on this scaffold and a culture collection of fifty-eight *Ralstonia pickettii *and *Ralstonia insidiosa *strains were analysed for the presence of the element.

**Results:**

Comparative sequence analysis of bacterial genomes has revealed the presence of a number of uncharacterised Tn*4371*-like ICEs in the genomes of several β and γ- Proteobacteria. These elements vary in size, GC content, putative function and have a mosaic-like structure of plasmid- and phage-like sequences which is typical of Tn*4371*-like ICEs. These elements were found after a through search of the GenBank database. The elements, which are found in *Ralstonia*, *Delftia*, *Acidovorax*, *Bordetella*, *Comamonas, Acidovorax*, *Congregibacter*, *Shewanella*, *Pseudomonas Stenotrophomonas*, *Thioalkalivibrio *sp. HL-EbGR7, *Polaromonas*, *Burkholderia *and *Diaphorobacter *sp. share a common scaffold. A PCR method was designed (based on the Tn*4371*- like element detected in the *Ralstonia pickettii *12J genome) to detect and characterise new elements of this type.

**Conclusion:**

All elements found in this study possess a common scaffold of core genes but contain different accessory genes. A new uniform nomenclature is suggested for ICEs of the Tn*4371 *family. Two novel Tn*4371*-like ICE were discovered and characterised, using the novel PCR method described in two different isolates of *Ralstonia pickettii *from laboratory purified water.

## Background

Integrative Conjugative Elements (ICEs) carry functional modules involved in their conjugative transfer, chromosomal integration and for control of expression of ICE genes [1]. ICEs are maintained in their host via site-specific integration and establishment at a unique site or sites in their host [2-7]. ICEs have been discovered in the genomes of various low G+C Gram-positive bacteria, various α, *β*- and *γ*-Proteobacteria, and *Bacteroides *species [8]. The first ICE found was Tn*916 *from *Bacteroides *species [8].

One of the best models of ICEs is a family of elements called the R391\SXT family that are found in *γ*-Proteobacteria. These are interesting elements as over 25 have been found to date in organisms spread across the world. They share a common core scaffold of genes related to integration, excision, transfer and regulation. Different elements can possess different fitness determinants such as antibiotic resistances, heavy metal resistances, and error-prone DNA repair systems [9].

Tn*4371 *is a 55-kb ICE, which allows its host to degrade biphenyl and 4-chlorobiphenyl. It was isolated after mating between *Cupriavidus oxalaticus *(*Ralstonia oxalatica*) A5 carrying the broad-host-range conjugative plasmid RP4 and *Cupriavidus metallidurans *(*Ralstonia metallidurans*) CH34. Selection was applied for transconjugants that expressed the heavy metal resistances from CH34 and grew with biphenyl as a sole source of carbon and energy [10]. The transconjugants carried an RP4 plasmid with a 55-kb insert near its tetracycline resistance operon. The insert was shown to transpose to other locations and hence was called Tn*4371 *[10-12]. Tn*4371 *has been sequenced [13] and closely related elements have been found in the genome sequences of a number of bacteria including *Ralstonia solanacearum *GMI1000, a phytopathogen from French Guyana [14], *Cupriavidus metallidurans *CH34, a heavy metal resistant bacteria from Belgium [15], *Erwinia chrysanthemi *3937, aphytopathogen [16] and *Azotobacter vinelandii *AvOP, a nitrogen-fixing bacterium isolated from soil in the USA [13,17]. None of these other elements possessed the biphenyl and 4-chlorobiphenyl degradation genes.

The Tn*4371*-like ICEs characterised to date are mosaic in structure consisting of Ti-RP4-like transfer systems, an integrase region, plasmid maintenance genes and accessory genes [13]. All the characterised elements integrate into sites on the bacterial genomes with a conserved 5'-TTTTTCAT-3' sequence, termed the *att*B site [11]. Tn*4371 *transposition most likely involves a site-specific integration/excision process, since the ends of the element can be detected covalently linked as a transfer intermediate [11,13]. Integration is catalysed by a tyrosine based site specific recombinase related to bacteriophage and ICE family integrases [18].

A small number of putative ICEs have been discovered following sequence analyses of genomes of various low G+C Gram-positive bacteria [19], various *α*, *β*- and *γ*-Proteobacteria [20-22], and *Bacteroides *species [23].

We now report the discovery and comparative analysis of a number of novel uncharacterised Tn*4371*-like ICEs from several different bacterial species. These elements are also mosaics of plasmid and other genes and posses a common scaffold with apparent hotspots containing insertions of different presumably adaptive genes. Using sequences from the common scaffold a PCR method was developed to discover and characterise new Tn*4371*-like ICEs in different bacteria. Here we report on the use of this method to discover and characterise two new Tn*4371*-like ICEs in *Ralstonia pickettii *strains isolated from a purified water system. Furthermore we propose a uniform nomenclature for newly discovered ICEs of the Tn*4371 *family

## Results and Discussion

### Bioinformatic analysis of Tn*4371*-like ICEs

Using bioinformatic analysis tools, searches of the genome databases for elements similar to the Tn*4371 *element were carried out using the original Tn*4371 *sequence as a probe. The method used was similar to that used to detect novel members of the R391/SXT family of ICEs in Enterobacteriaceae [22]. In this study novel unreported ICEs closely related to Tn*4371 *were discovered in the genome sequences of several different bacteria including the β-proteobacteria, two elements in *Delftia acidovorans *SPH-1, and a single element *Comamonas testosteroni *KF-1, *Acidovorax avenae *subsp. citrulli AAC00-1, *Bordetella petrii *DSM12804, *Acidovorax *sp. JS42, *Polaromonas naphthalenivorans *CJ2 plasmid pPNAP01, *Burkholderia pseudomallei *MSHR346 and *Diaphorobacter *sp. TPSY [Table 1]. Novel elements were also found in the γ-proteobacteria *Congregibacter litoralis *KT71, *Shewanella *sp. ANA-3, *Pseudomonas aeruginosa *2192, *Pseudomonas aeruginosa *PA7, *Pseudomonas aeruginosa *PACS171b, *Pseudomonas aeruginosa *UCBPP-PA14, *Stenotrophomonas maltophilia *K279a, *Thioalkalivibrio *sp. HL-EbGR7 [Table 2]. The element in *Bordetella petrii *DSM12804 was previously identified but not analyzed in a paper by Lechner *et al*., [24]. The elements found in *Delftia acidovorans *SPH-1, *Comamonas testosteroni *KF-1 and *Bordetella petrii *DSM12804 were also partially characterised along with further information on the elements in *Cupriavidus metallidurans *CH34 in a paper by Van Houdt *et al*., [25]. Geographically all these bacteria were found in different locations in both Europe and the Americas and were isolated from many different environments including activated sludge, polluted water and clinical situations [Table 1 and 2]. All elements contained different inserts [containing accessory genes] in the core backbone except for those found in *Delftia acidovorans *SPH-1 and *Comamonas testosteroni *KF-1. The size of the newly discovered elements varied from 42 to 70 Kb and the GC content from 59 to 65% [Table 1 and 2].

**Table 1:** Size and %GC Content, accessory Genes contained in and the location and environment of isolated strains containing Tn*4371*-like ICEs from*β*-Proteobacteria

Tn*4371*-like Elements	Size	%GC Content	Location	Environment	Accessory Genes	Reference	Name	Accession Number
*Ralstonia pickettii *12J	54121 bp	64.63	USA	Copper-contaminated sediment from a lake	Lipid metabolism	[74]	ICE_Tn*4371*_*6033*	CP001068

*Acidovorax avenae *subsp. citrulli AAC00-1	59844 bp	63.12	USA	Watermelon	Insertion Sequences metabolism	[75]	ICE_Tn*4371*_*6036*	NC_008752

*Delftia acidovorans *SPH-1	57901 bp	63.66	Germany	Activated sludge	*czc *metal resistance pumps	[76]	ICE_Tn*4371*_*6037*	NC_010002

*Comamonas testosteroni *KF-1	52455 bp	63.77	Switzerland	Activated sludge	*czc *metal resistance pumps	[76]	ICE_Tn*4371*_*6038*	NZ_AAUJ0100000

*Acidovorax *sp. JS42	53489 bp	62.88	USA	Groundwater	Multidrug resistance pump Insertion Sequences	[77]	ICE_Tn*4371*_*6039*	NC_008782

*Bordetella petrii* DSM12804	47191 bp	63.73	Germany	River sediment	Aromatic compounds metabolism	[78]	ICE_Tn*4371*_*6040*	NC_010170

*Burkholderia pseudomallei *MSHR346	49278 bp	62.21	Australia	Melioidosis patient	metabolism	N/A	ICE_Tn*4371*_*6064*	CP001408

*Polaromonas naphthalenivorans *CJ2 plasmid pPNAP01	70106 bp	62.89	USA	Coal-tar-waste contaminated site	Biphenyl degradation	[79]	ICE_Tn*4371*_*6065*	CP000530

*Diaphorobacter *sp. TPSY	49020 bp	65.30	USA	Soil	*czc *metal resistance pumps	[80]	ICE_Tn*4371*_*6066*	CP001392

*Delftia acidovorans *SPH-1	66755 bp	64.94	Germany	Activated sludge	Various types of metal resistance pumps	[76]	ICE_Tn*4371*_*6067*	NC_010002

**Table 2:** Size and %GC Content, accessory Genes contained in and the location and environment of isolated strains containing Tn*4371*-like ICEs from *γ*-Proteobacteria

Tn*4371*-like Elements	Size	%GC Content	Location	Environment	Accessory Genes	Reference	Name	Accession Number
*Shewanella *sp. ANA-3	45233 bp	59.43	USA	Arsenate treated wood pier	Multidrug resistance pump	[81]	ICE_Tn*4371*_*6034*	NC_008577

*Congregibacter litoralis *KT71	50661 bp	59.52	North Sea	Ocean-surface water	RND type multidrug efflux pump	[82]	ICE_Tn*4371*_*6035*	NZ_AAOA01000008

*Pseudomonas aeruginosa *2192	48538 bp	62.62	USA	Cystic fibrosis patient	RND type multidrug efflux pump	[83]	ICE_Tn*4371*_*6041*	NZ_AAKW01000024

*Pseudomonas aeruginosa *PA7	55287 bp	52.38	Argentina	Clinical wound isolate	Multiple antibiotic resistance genes Potassium transporter system	[84]	ICE_Tn*4371*_*6042*	NC_009656

*Stenotrophomonas maltophilia *K279a	43509 bp	62.76	UK	Blood infection	Multidrug resistance pump	[85]	ICE_Tn*4371*_*6068*	AM743169

*Pseudomonas aeruginosa *UCBPP-PA14	43172 bp	65.55	USA	Burn patient	*czc *metal resistance pumps	[86]	ICE_Tn*4371*_*6069*	CP000438

*Pseudomonas aeruginosa *PACS171b	42156 bp	64.12	USA	Cystic fibrosis patient	Arsenate resistance pumps	[87]	ICE_Tn*4371*_*6070*	EU595746

*Thioalkalivibrio *sp. HL-EbGR7	42540 bp	64.95	Unknown	bioreactor removing sulfide from biogas	Potassium transporter system	[88]	ICE_Tn*4371*_*6071*	CP001392

### Characterisation of Tn*4371*-like ICEs in whole genome sequences

**Figure 1:**
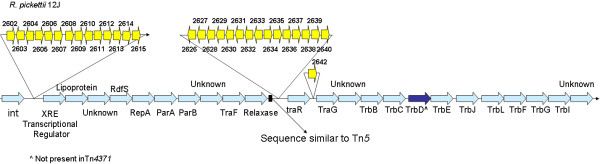
**Common core scaffold of Tn*4371*-like ICEs (in blue) and above inserted genes present in *R. pickettii *ICE_Tn*4371*_*6033 *(in yellow)**.

The core structure conserved amongst known Tn*4371*-like ICEs is presented in Fig. 1. At the *attR *end of the elements a putative *int *gene [that bears similarities to tyrosine based site-specific recombinases historically called phage-like integrases [20], possessing the R-H-R-Y tetrad] is found [Additional file 1]. A phylogenetic study was carried out on all available Tn*4371*-like *int *genes and tyrosine recombinases from phages and other ICEs. The phylogenetic tree can be seen in Additional file 2. These Tn*4371*-like *int *genes grouped with the *int *genes of ICE *Hin1056*, an ICE from *Haemophilus influenzae *and from phages related to the P22 phage. The *int *gene was found in all characterised elements and was followed by nonconserved ORFs which differed from element to element. These ORFs include putative DNA helicases and nucleases, proteins with β-lactamase domains, similar to RadC DNA repair proteins, putative reductases, transposases of insertion sequences, putative ubiquitin-activating enzymes, putative transcriptional regulators and many different hypothetical proteins whose functions are unknown [Fig. 1, Additional file 3]. These ORF's were found in differing arrangements in each of the different elements. *Polaromonas naphthalenivorans *CJ2 plasmid pPNAP01 contained biphenyl degradation genes in this area of the element and these genes are similar to those found in the original Tn*4371 *element but are found in a different part of the element. *Pseudomonas aeruginosa *PACS171b and the second *Delftia acidovorans *SPH-1 element have an arsenate resistance system located in this region. This system is related to the ars system, and has the genes *arsH*, *arsC, arsB *and *arsA *in the operon in this bacterium. The function of *arsH *is unknown; however it is necessary for resistance to arsenic in the *Yersinia enterocolitica *virulence plasmid pYV [27]. The *arsC *gene encodes a soluble arsenate reductase which reduces intracellular arsenate to arsenite for efflux from the cell [28]. The *arsA *gene codes for a unique ATPase which binds to the ArsB membrane protein forming an anion transporting arsenite pump [28]. The *arsD *gene encodes an inducer independent regulatory protein which controls the upper level of operon expression [29]. The second *Delftia acidovorans *SPH-1 element has genes related to the *Mer *(Mercury Resistance) operon: *merR, merT, merP and merA*. The *merR *gene controls regulation of the operon, *merT *and *merP *transport of the mercury ions and *merA *reduction of the mercury ions [30]. This region also contains a predicted *czc *[Cd/Zn/Co] efflux system [31,32]. *Czc *mediates the inducible resistance to Co^2+^, Zn^2+ ^and Cd^2+^, the protein products of gens *czcA, czcB *and *czc *form a membrane-bound protein complex catalysing an energy dependant efflux of these three metal ions [33].

Following the integrase gene two conserved genes [ORF00013 and ORF00014 in Tn*4371*] were present in most elements except those in *C. litoralis *KT71 and *Shewanella *sp. ANA-3. These ORF's are related to proteins encoded by genes located near the transfer origin of *Escherichia coli *F plasmid [Q9WTE4 and Q9S4W2]. Although the function of the first protein is unknown, the second shows similarity to ParB-like nucleases initially identified as a critical element in the faithful partitioning of plasmid DNA during cell division in the absence of selection pressure [34,35]. Subsequently, a number of similar proteins have been identified in prokaryotes and archea which carry out the function of segregation of genomic DNA during cell division. ParB homologs are present in almost all eubacteria chromosomes [36].

The next region on all elements contains proteins similar of the XRE [Xenobiotic Responsive Element] family of transcriptional regulators, a putative lipoprotein with a DNA binding domain and a protein of unknown function. The XRE family behave as lambda repressor-like proteins associated with different phages, including *Staphylococcus aureus *phage phi 11 [37] and the *Bacillus subtilis *defective prophage PBSX [[38], Fig. 1]. Two different homologues of the XRE were found in different elements one related to that found in the original Tn*4371 *element (*R. pickettii *12J, *D. acidovorans *SPH-1, *A. avenae subsp. citrulli *AAC00-1, *C. testosteroni *KF-1 and *Acidovorax *sp. JS42, *C. litoralis *KT71, *Shewanella *sp. ANA-3, *P. aeruginosa *2192 and *P. aeruginosa *PA7, *P. aeruginosa *PACS171b, *Thioalkalivibrio *sp. HL-EbGR7 and *B. pseudomallei *MSHR346). A different XRE was found in the remaining elements: *B. petrii *DSM 12804, *S. maltophilia *K279a, *P. aeruginosa *UCBPP-PA14, *Diaphorobacter *sp. TPSY, *P. naphthalenivorans *CJ2 plasmid pPNAP01 and the second element of *Delftia acidovorans *SPH-1.

Following on from the XRE transcriptional regulators, a protein [ORF00035 of Tn*4371*] was found with similarity to the RdfS excisionase [CAD31514] of ICE*Ml*Sym^R7A^, the symbiosis island of *Mesorhizobium loti *R7A [39]. Most excisionases, also called recombination directionality factors [RDF's], share a number of conserved features: they are small [usually <100 amino acids] DNA-binding proteins, that are typically basic with the majority of known RDFs having isoelectric points in the range of pH 8-10 [40]. The size of the ORF00035 protein homologues found in this comparative analysis ranged from 89-98 aa [amino acid] and had pI's ranging from 8.14 to 9.59. BlastP scores showed approximately 50% aa identity with the ICE*Ml*Sym^R7A ^RdfS, over approximately 55 aa for all of the putative RdfSs discovered in this study [Fig. 1]. No excisionase was found in the second *Delftia acidovorans *SPH-1 element. The location of this ORF is also of interest as usually excisionases are found close to the integrase gene in most ICEs particularly the SXT/R391 family [41].

The next sequence region of the elements contained plasmid-related genes whose predicated products were related to the RepA [replication] protein of *Pseudomonas *plasmid pVS1 [BAA96327, 42], plasmid pMLb of *M. loti *MAFF303099 [NP_109574, 43] and plasmid pEMT8 [CAC94910, 44]; this gene maybe involved in replication of the element [13]. The ParA partition protein of the type Ib family [45] and its associated ParB protein was also found but in all cases the ParB was truncated. Rep and Par proteins have been proposed to act as a stabilisation system for the maintenance of mobile elements in bacterial genomes [19,36], similar to the toxin-anti-toxin system encoded by ORFs s044 and s045 of the SXT-ICE [46]. Qui *et al*. found that the *P. aeruginosa *ICE PAPI-1 contains a homologue of the plasmid and chromosome partitioning gene *soj *(*parA*). They demonstrated that deletion of the *soj *homologue from PAPI-1 resulted in complete loss of PAPI-1 from *P. aeruginosa*. The mechanism by which the Soj protein promotes PAPI-1 maintenance remains to be elucidated [47]. Similar genes to *soj *have been found in ICE *Hin1056 *and ICEA [20,48]. This region was followed by an ORF encoding a conserved hypothetical protein [ORF00040 in Tn*4371*] whose function is unknown [Fig. 1].

This sequence is followed by a region containing transfer like proteins, the first being a putative conjugation protein TraF related to the pilus assembly proteins of IncP plasmids. This TraF protein is a protease that acts upon the pilus assembly protein TrbC [49]. The second is a putative relaxase-like protein [ORF00041 in Tn*4371*] that has similarity to the VirD2 protein of Ti plasmids and to the RlxS [relaxase, CAD31511] of ICE*Ml*Sym^R7A^. Transfer and maintenance of ICE*Ml*Sym^R7A ^in cells has been shown to be dependent on the relaxase protein RlxS [[39], Fig. 1]. A relaxase, usually encoded by the plasmid, recognizes *oriT*, makes a single-strand DNA break (a nick) in *oriT*, and covalently attaches to the 5' end of the nicked DNA strand via a phosphotyrosyl linkage. No helicase domain was found in examining the protein so this indicates that the element may use leading-strand DNA synthesis (rolling-circle replication) from the nicked 3' end to promote strand displacement and single-strand DNA transfer [50,51].

Following the putative relaxase-like protein is a variable region encoding a number of different ORFs, which vary from element to element; these genes encode putative antibiotic genes, heavy metal resistance pumps and degradative and metabolic enzymes which may have originated by transposition into the element. The sequence between the putative relaxase gene and the first gene of the variable region, in all elements, is similar to the sequence of an area of Tn*5 *[U00004] indicating that the diversity in this region maybe due to one or a number of Tn*5 *mediated insertion events. This variable region in the novel ICE in *R. pickettii *12J encodes a putative set of lipid metabolising genes [Fig. 1]. These are closely related to genes from *Pseudomonas putida *W619 [NZ_AAVY01000010.1] and from the pREC1 plasmid from *Rhodococcus erythropolis *PR4 [NC_007486] [52]. *A. avenae *subsp. citrulli AAC00-1 contained insertion sequences and homologues to general metabolism proteins whose exact functions are unknown. *D. acidovorans *SPH-1 and *C. testosteroni *KF-1 contain a predicted *czc *[Cd/Zn/Co] efflux system [31,32] in their variable regions. The novel element in *Acidovorax *sp. JS42 contains genes that show similarity to a multidrug resistance pump and insertion sequences [InterPro Scan] in this region. In the variable region in *B. petrii *DSM 12804 there are various proteins that are putatively involved in degradation, however their exact function is unknown. *Burkholderia pseudomallei *MSHR346 has genes that are putatively involved in xenobiotic metabolism; however again their exact function is unknown. *Polaromonas naphthalenivorans *CJ2 plasmid pPNAP01 contains a putative antibiotic resistance pump and metabolism proteins whose role have not been identified. *Diaphorobacter *sp. TPSY contains a predicted *czc *[Cd/Zn/Co] efflux system similar to those in *D. acidovorans *SPH-1 and *C. testosteroni *KF-1. The second *D. acidovorans *SPH-1 contains a copper resistance system Cop related to that of *Pseudomonas syringae*. The genes in this system are laid out in the following order *copSR copABFCD. copSR *is a two-component signal transduction system, which is required for the copper-inducible expression of copper resistance [53]. CopA and CopC are abundant periplasmic copper binding proteins, and CopB is associated with copper accumulation in the outer membrane. No specific function for CopD has been determined yet [54]. CopF is involved in the cytoplasmic detoxification of copper ions [55]. In the novel element associated with *Shewanella *sp. ANA-3 the variable region encodes genes that shares similarities with a chloramphenicol efflux pump [InterPro Scan]. *C. litoralis *KT71 and *P. aeruginosa *2192 have a putative resistance nodulation division [RND] type multidrug efflux pump related to the *mex *system of *P. aeruginosa *[56] and the *oqx *system of *E. coli *plasmid pOLA52 [57] encoded. Apart from antibiotics, the broad substrate range of the Mex efflux systems of *P. aeruginosa *also includes organic solvents, biocides, dyes, and cell signalling molecules [58]. In the ICE of *P. aeruginosa *PA7 this variable region encodes homologs of genes for antibiotic resistance including neomycin/kanamycin resistance, bleomycin resistance, and streptomycin resistance related to the antibiotic resistance genes from Tn*5 *[U00004]. There are also a set of genes with similarity to the *kdpFABC *system. The KdpFABC complex acts as a high affinity K^+ ^uptake system. In *E. coli*, the complex is synthesized when the constitutively expressed low affinity K^+ ^uptake systems Trk and Kup can no longer meet the cell's demand for potassium due to external K^+ ^limitation Altendorf et al., 1992 K. Altendorf, A. Siebers and W. Epstein, The KDP ATPase of *Escherichia coli*, *Ann. NY Acad. Sci*. **671 **(1992), pp. 228-243. **Full Text**via CrossRef|View Record in Scopus Cited By in Scopus (22)[59]. *Stenotrophomonas maltophilia *K279a had a putative Major Facilitator Superfamily (MFS) efflux pump that usually function as specific exporters for certain classes of antimicrobial agents. This is related to the *emrAB *system from *E. coli *[60]. *P. aeruginosa *UCBPP-PA14 has a predicted *czc *[Cd/Zn/Co] efflux system similar to those in *D. acidovorans *SPH-1 and *C. testosteroni *KF-1. *P. aeruginosa *PACS171b contains a homolog of UspA- the Universal Stress Protein. The UspA protein is important for survival during cellular growth arrest in *E. coli*, but the exact physiological role of the protein is unknown [61]. *Thioalkalivibrio *sp. HL-EbGR7 has a set of genes with approximately 88% aa identity to the putative KdpFABC system in *P. aeruginosa *PA7. This variability is suggestive that this region may be a hotspot for insertion or recombination where insertion clearly does not disrupt or affect the expression of neighbouring genes. The variation in predicted gene function, size and lack of homology between elements is suggestive of this region contributing a number of different adaptive traits to hosts containing these ICEs.

Following this variable region is encoded a putative transcriptional regulator protein TraR and a homologue of the type IV coupling protein TraG [similar to those in IncP plasmids]. TraG is responsible for DNA transfer during conjugation and is a putative DNA binding protein [62]. Interestingly the gene order of this region and the order of genes preceding it are also suggestive of an insertion [of the variable region just discussed] into a primordial transfer module.

The putative DNA binding gene *traG *is followed by a group of genes encoding proteins [TrbBCDEJLFGI] with similarity to the mating-pair formation [mpf] apparatus or type IV secretion system closely related to IncP and Ti plasmids. This system presumably mediates the DNA transfer of the ICE to recipient cells [63,64]. These genes show similarity to those required for conjugative transfer of the *Agrobacterium *Ti plasmid, pNGR234a and RP4, except that two genes, *trbK *and *trbH*, found on these plasmids are missing [65]. In the Tn*4371*-like elements the gene order was trb *BCDEJLFGI *in all the characterised elements found in this study and similar to the molecular organisation in ICE*Ml*Sym^R7A ^[[19], Fig. 1]. The TrbB, TrbC, TrbE, TrbG, and TrbL proteins are involved in the creation of the mpf apparatus, TrbC is involved in pilus formation and TrbE displays ATPase activity [65].

The novel ICEs detected in this study are integrated into various locations in the genomes of the host bacteria where they were discovered. In *Acidovorax *sp. JS42 other partial copies of Tn*4371*-like elements were also found in addition to the full element reported here. Two elements were discovered and characterised in *D. acidovorans *SPH-1. A further partial element was found in *B. petrii *this however lacked the *int*_Tn*4371 *_gene. This situation is similar to that found in *R. metallidurans *CH34 and indicates that duplication or multiple insertions of the elements occur in bacteria. Near complete copies of Tn*4371*-like elements were also found in *Burkholderia ambifaria *AMMD and *Burkholderia multivorans *ATCC17616, where both were found to lack the Tn*4371*-like integrase gene suggesting that the elements may no longer be mobile. New elements were also found in *Ralstonia solanacearum *MolK2 and a second element in *Diaphorobacter *sp. TPSY, these share similarities in the stabilisation and transfer regions of the element to Tn*4371*-like elements but they have a different integrase region not related to the *int*_Tn*4371 *_gene.

**Figure 2:**
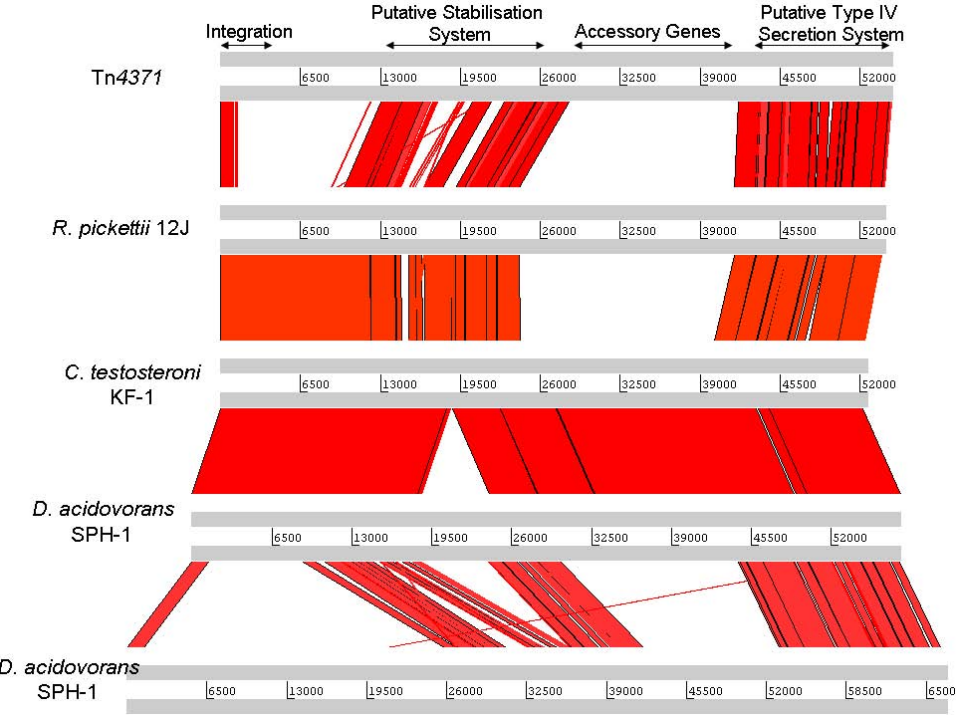
**Use of the Artemis comparison tool to analysis Tn*4371*-like ICE sequences of Tn*4371, R. pickettii *12J, both elements from *D. acidovorans *SPH-1 and *C. testosteroni *KF-1**. All ICEs analysed shared extensive sequence homology, and general gene order. Arrows on top delimit the functional regions whose order is well conserved in all Tn*4371*-like ICEs.

**Figure 3:**
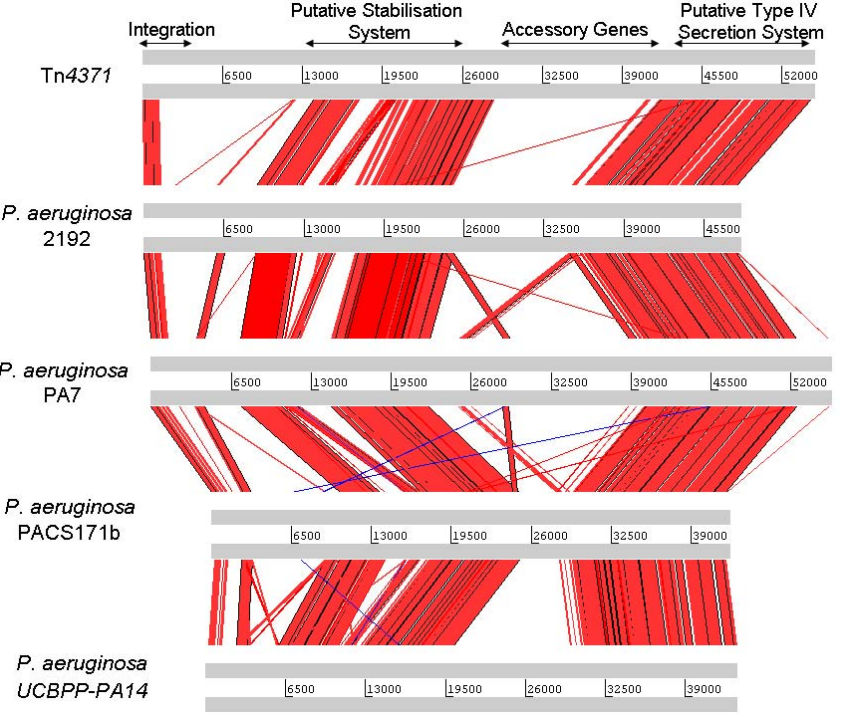
**Use of the Artemis comparison tool to analysis Tn*4371*-like ICE sequences of Tn*4371*, *P. aeruginosa *2192, *P. aeruginosa *PA7, *P. aeruginosa *UCBPP-PA14 and *P. aeruginosa *PACS171b**. All ICEs analysed shared extensive sequence homology, and general gene order. Arrows on top delimit the functional regions whose order is well conserved in all Tn*4371*-like ICEs.

**Figure 4:**
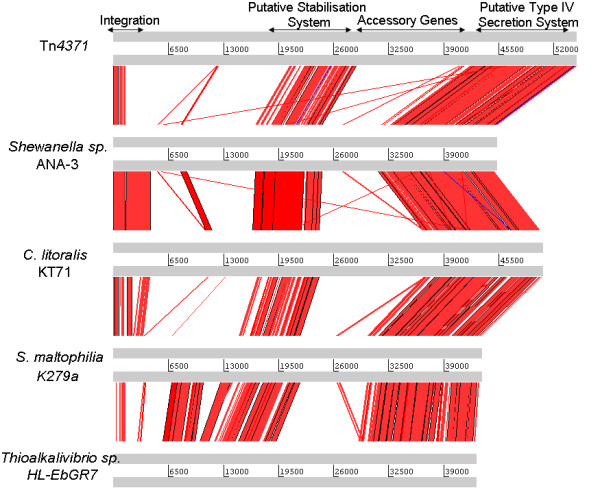
**Use of the Artemis comparison tool to analysis Tn*4371*-like ICE sequences of Tn*4371, Shewanella *sp. ANA-3, *C. litoralis *KT71, *S. maltophilia *K279a and *Thioalkalivibrio *sp. HL-EbGR7**. All ICEs analysed shared extensive sequence homology, and general gene order. Arrows on top delimit the functional regions whose order is well conserved in all Tn*4371*-like ICEs.

**Figure 5:**
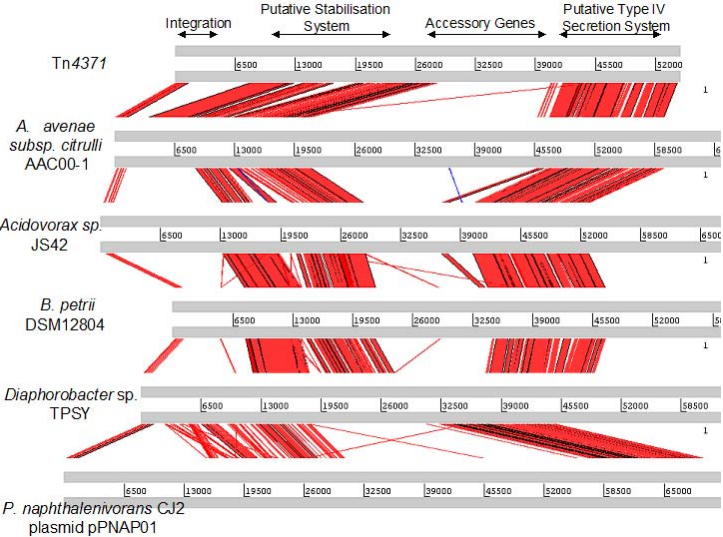
**Use of the Artemis comparison tool to analysis Tn*4371*-like ICE sequences of Tn*4371*, *A. avenae *subsp. citrulli AAC00-1, *Acidovorax *sp. JS42, *B. petrii *DSM12804, *Diaphorobacter *sp. TPSY and *P. naphthalenivorans *CJ2 plasmid pPNAP01**. All ICEs analysed shared extensive sequence homology, and general gene order. Arrows on top delimit the functional regions whose order is well conserved in all Tn*4371*-like ICEs.

All of the elements reported here [Table 1 and 2] appear to share a common scaffold or backbone that is approximately 24 kb in size containing a 1.5 kb integrase gene; an 8.5 kb replication/stability gene cluster and a 14 kb conjugal transfer/mating pair formation cluster [Fig. 1]. A visual representation of this can be seen in Figs. 2, 3, 4 and 5 where the various sequences were aligned for comparison, the core scaffold identified and 'adaptive' genes highlighted which vary from element to element.

Bioinformatic comparisons were performed between the genes that make up the core scaffold region of the ICE and these ranged from the highly conserved *traG *gene, with 84 to 96% aa identity, *trbE *gene, with 76 to 94% aa identity, and the *parA *gene, with 90 to 97% aa identity, to the less-conserved *traR *gene, with 53 to 84% aa identity. On average the genes that we ascribed to the core showed > 75% aa identity and were also related by gene order. All gene numbers and a basic description of the genes are included in Additional file 3.

### Defining the Tn*4371 *family of ICEs and nomenclature

These elements have been classed as ICEs as we believe at this moment in time this is the best terminology currently available. They follow all the criteria of ICEs having integration and transfer modules, possessing an excisionase gene and having genes and gene layout (*rdfS*, *rlxS *and the *trb *genes) similar to other ICEs namely ICE*Ml*Sym^R7A^. The original element can also excise from bacterial chromosome and form a circular intermediate [9], however the element has not been shown to transfer between different bacteria, and this could be due to the original element lacking the *trbD *gene [13].

Although the elements identified in this study are not identical, they share a similar core backbone that, in our view, warrants their inclusion into the Tn*4371 *ICE family. All encode a related integrase, related maintenance and transfer genes and the gene order of homologous genes are similar, if one were to remove variable inserted regions which differ from element to element. We propose that any ICE that encodes an integrase gene closely related to *int*_Tn*4371*_, defined as over 70% protein homology and that has similar maintenance and transfer genes be considered part of the Tn*4371 *family of ICEs.

Given the number of Tn*4371*-like elements discovered in this study, it seems sensible to name newly described ICEs of the Tn*4371 *family with a uniform nomenclature. We propose adapting the system used for naming transposons described by Roberts *et al*., [66]. This system is a website http://www.ucl.ac.uk/eastman/tn/ based system which assigns Tn numbers in sequence e.g. Tn*6033*, Tn*6034*, etc and the elements were then called ICE_Tn*4371*_*6033*, ICE_Tn*4371*_*6034*, etc to distinguish that they are ICEs of the Tn*4371 *family. The names assigned to the elements discovered in this study are listed in Table 1 and 2. This system was chosen as other systems such as that used by Burrus *et al*., [8] for naming members of the SXT\R391 family of ICEs are not regulated and can differ between laboratories leading to confusion.

### Tn*4371*-like ICE detection and molecular characterisation

Following the discovery of the widespread nature of Tn*4371*-like ICEs in the genomes of many new organisms, PCR primers were designed to amplify important genes of the core scaffold to aid in the rapid identification of new Tn*4371*-like elements. We tested this on a culture collection of fifty-eight *Ralstonia pickettii *and *Ralstonia insidiosa *strains from various environments and geographic locations. The PCR primers were based on conserved consensus sequences of core genes identified from all the elements identified in this study and those reported previously.

**Figure 6:**
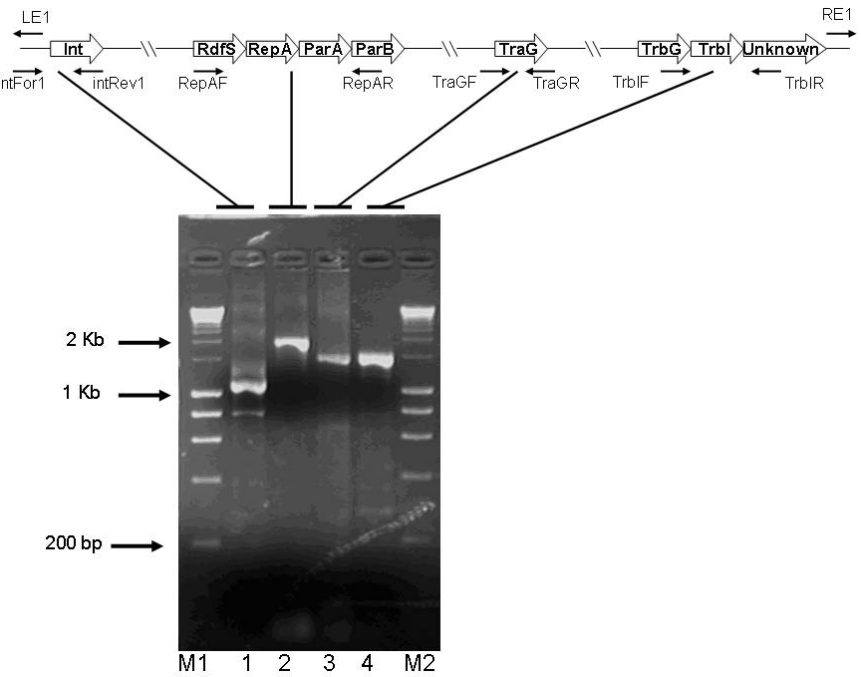
**Amplification of genes of the putative Tn*4371*-like ICE **ICE_Tn*4371*_*6043 ***in *Ralstonia pickettii *strain ULM001 (a laboratory purified water isolate)**. A scheme of the amplified genes is shown above the 0.7% agarose gel of the PCR products generated with the primers listed in Table 2. Open white arrows denote ORFs of the *Ralstonia pickettii *ICE, and small black arrows represent the relative location of primers. Lanes M1 and M2 contain 200-10000 bp molecular size markers (Bioline Hyperladder I), respectively. The lanes and the product sizes are as follows: Lane 1, *int *gene and flanking bases (1035 bp); Lane 2 *RepA *gene (1657 bp), Lane 3 *traG *gene (1483 bp); Lane 4 *trbI *gene (1597 bp).

The results in Fig. 6 show the genes encoding a putative integrase [*int*], the putative stabilisation system [*repA, parA, parB*], a homologue to the DNA transfer protein [*traG*] and a putative pilus assembly and synthesis protein [*trbI*]. DNA sequencing of the four amplicons in the tester strains demonstrated that Tn*4371*-like sequences exist in the genome of *R. pickettii *ULM001. While this data clearly demonstrates the presence of Tn*4371*-like elements in tester strains the possibility of multiple elements in such strains cannot be excluded, although out sequencing of resulting amplicons is suggestive of only one element.

Three of the fifty-eight *Ralstonia *isolates, ULM001, ULM003 and ULM006 [which were laboratory purified water isolates from different locations in France] showed positive amplification for *int*_Tn*4371 *_integrase gene when tested with the intFor1 and intRev1 primer pair in PCR amplification [Table 3]. Sequencing revealed that the ULM001 *int *gene showed 85% and 99% nucleotide identity to the Tn*4371 int *gene and ICE_Tn*4371*_*6033 int *gene, respectively. The RepAF and RepAR primers also amplified the *repA *gene and the *parA *gene in ULM001, ULM003 and ULM006. Sequencing these amplicons revealed that in ULM001 the repA and parB genes were present and showed 88% and 99% nucleotide identity to the *RepA *and *ParA *genes from Tn*4371 *and ICE_Tn*4371*_*6033 *respectively. A *traG*_Tn*4371 *_homolog was also detected in ULM001, ULM003 and ULM006 following PCR amplification. Sequencing revealed that the ULM001 *traG*_Tn*4371 *_gene showed 91% and 89% nucleotide identity to *traG *from Tn*4371 *and ICE_Tn*4371*_*6033 *respectively. TrbIF and TrbIR primers were used to amplify the *trbI *gene in ULM001 and ULM003 while no amplification occurred in ULM006. Sequencing showed that the ULM001 amplicon was a homolog, which had 88% and 99% nucleotide identity to the *trbI *gene from Tn*4371 *and ICE_Tn*4371*_*6033 *respectively. The absence of a *trbI *gene amplicon in ULM006 may indicate a deleted gene or truncated element in this strain. The use of these primer sets has thus revealed the presence of two new elements, which can then be further characterised. The ICEs detected in this study from *Ralstonia pickettii *were named ICE_Tn*4371*_*6043 *and ICE_Tn*4371*_*6044 *using the nomenclature system described above, a general map of the elements can be seen in Fig. 6.

**Table 3:** Primers used to map Tn*4371*-like ICEs

Genes	Size (bp)	Primers	T_m_(°C)	*R. pickettii *12J Position		**Accession no**.
					
				Start	Stop	
CirIm	~220	RE1 GCATGGAAGACTTGACAG LE1 GAGCTTGAGTTTTGCCACG	54	N\A	N\A	FM244490

int	1035	intFor1 TTTCATTTCACCATGACTCCAG intRev1 GAGAGCAGTCGATAGGCTTCC	61.7	2715201	2716235	FM244486

RepA, ParA ParB	1657	RepAF GAGACTACCAGCGCCTCAAG RepAR ACGTGTTCATGAGGACTTCTCC	55	2734598	2736255	FM244487

traG	1483	traGF GTTCGAGTGGTGGTTCTTCTTC traGR GAAATTGCTGTCCGCGTAGTAG	61	2757179	2758661	FM244488

trbI	1597	trbIF AACTGACCATGAGCCAGGAC trbIR AAAGCTCCTCAAAAGCGAAAG	62	2767516	2769113	FM244489

### The *attL *and *attR *region of Tn*4371 *ICEs

**Figure 7:**
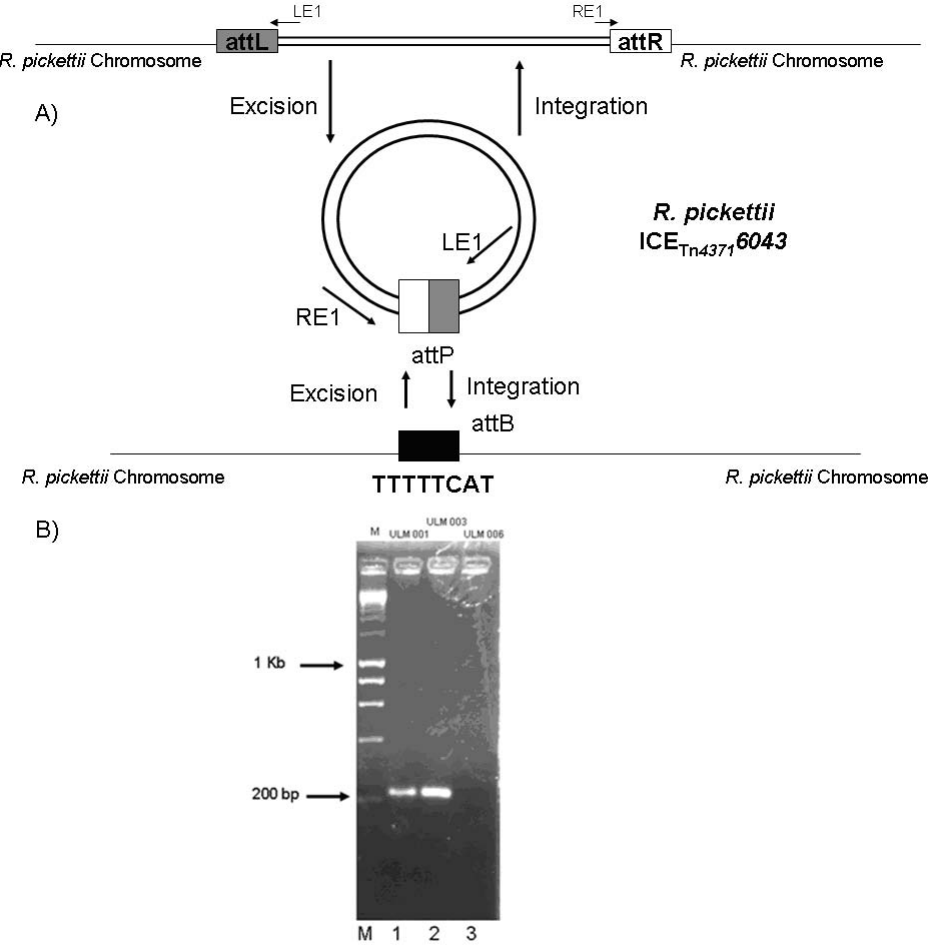
**A) Schematic representation of Tn*4371 *excision and insertion into the *R. pickettii *chromosome**. Primer LE1 and RE1 are the primers for detection of the circular form of the element. B) Agarose gel of *attP *of ICE_Tn*4371*_*6043 *and ICE_Tn*4371*_*6044*. Lanes M contains 200-10000 bp molecular size markers (Bioline Hyperladder I), Lane 1 ULM001, Lane2 ULM002, Lane 3 ULM006.

Analysis of hosts harbouring Tn*4371*-like elements indicated that integration occurred at an 8-bp *attB *site generating *attL *and *attR *element chromosomal junctions [[11], Fig. 7a]. An alignment of the first and last 200 bp of the elements analysed in this study with Tn*4371*-like element from previous studies showed the *attL *site had a sequence of TTTTC/TA/GT and *attR *had a sequence of TTTTC/TA/GT for some bacteria, while others had no direct repeats. These alignments can be seen in Additional file 4. The exact sequence of the direct repeat for each element is presented in Table 4. The absence of direct repeats in some of these elements may mean that they are no longer mobile. Tn*4371 *has been shown to excise from the RP4 plasmid in *Ralstonia eutropha *forming a circular extrachromosomal intermediate [[10], Fig. 7a] as a transfer intermediate. The strains in which we detected Tn*4371*-like elements were examined to see if they also excised forming extrachromosomal intermediates [CirIm] using a PCR assay that allowed amplification across the circular junction but which would not amplify if the element were integrated. Primer LE1 is specific to integrated Tn*4371*-like ICE DNA at the *attL *left-end where as primer RE1 is specific to integrated Tn*4371*-like ICE at the attR right-end [Fig. 7a, Table 3]. Both primers are oriented towards the Tn*4371*- like ICE junctions, and PCR product will be generated only if the respective left and right ends [*attL *and *attR *sites] excise from the chromosome and circularise [CirIm], reconstituting *attP *[attachment locus on the element]. A model of integration and excision of the ICE can be seen in Fig. 7a. PCR products of ~220-bp were obtained from ICE_Tn*4371*_*6043 *[ULM001] and ICE_Tn*4371*_*6044 *[ULM003] [Fig. 7b.], indicating that a circular extrachromosomal form of the element is present in these cells, while no PCR product was obtained from ULM006 [Fig. 7b]. The sequencing of the *attP *region of ICE_Tn*4371*_*6043 *gave an *attL *region of TTTTTCAT and an *attR *region of TACTTTTT. This rapid amplification across the circular *attP *junction can also be utilised for the rapid identification of Tn*4371*-like elements. It is possible that the PCR may have picked up tandems of the element if those happened to be intermediates in "transposition".

**Table 4:** Direct repeats at the ends of each element

Tn*4371*-like Elements	Direct repeats
*Ralstonia pickettii *12J	TTTTTCAT

*Shewanella *sp. ANA-3	TTTTTTAT

*Congregibacter litoralis *KT71	TTTTTTAT

*Acidovorax avenae *subsp. citrulli AAC00-1	TTTTTCAT

*Delftia acidovorans *SPH-1	TTTTTCAT

*Comamonas testosteroni *KF-1	TTTTTCAT

*Pseudomonas aeruginosa *2192	TTTTTTAT

*Pseudomonas aeruginosa *PA7	TTTTTTGT

*Stenotrophomonas maltophilia *K279a	TTTTTTGT

*Pseudomonas aeruginosa *PACS171b	TTTTTTAT

*Diaphorobacter sp*. TPSY	TTTTTCAT

*Delftia acidovorans *SPH-1	TTTTTCAT

*Acidovorax *sp. JS42	NP

*Bordetella petrii *DSM12804	NP

*Thioalkalivibrio sp*. HL-EbGR7	NP

*Burkholderia pseudomallei *MSHR346	NP

*Polaromonas naphthalenivorans *CJ2 plasmid pPNAP01	NP

*Pseudomonas aeruginosa *PA14	NP

NP, Not Present

## Conclusion

Tn*4371*-like ICEs are found in a wide range of γ-proteobacteria and β-proteobacteria from both clinical and environmental sources. These types of bacteria are known for their large metabolic repertoires and these elements could potentially be a source of acquisition of adaptive functions for these organisms. The discovery of the Tn*4371*-like ICEs in the *P. aeruginosa *strains, *S. maltophilia *K279a and *B. pseudomallei *MSHR346 are the first reports of these elements found in human pathogens. This along with the discovery of putative antibiotic resistance genes in their genomes indicates that these elements may have an impact in clinical situations. The discovery and characterisation of novel Tn*4371*-like elements as reported here adds significantly to the repertoire of such elements and helps define the core scaffold of such elements. It is clear that these elements are highly adaptable and may contribute significantly to the metabolic capabilities of their host. This study increases the knowledge available about these elements adding data on eighteen new elements to the five already known. A new nomenclature system for *Tn4371*-like elements was designed to avoid confusion in the naming of these elements. The primer system used to detect and characterise the Tn*4371*-like ICEs in *Ralstonia pickettii *ULM001 and ULM003 could be adapted and used for other bacterial species for the rapid screening of such elements.

## Methods

### Bacterial strains and growth conditions

The strains used in this study are shown in Table 5. All the strains were stored at -20°C in Nutrient Broth [Biolab, Budapest, Hungary] with 50% glycerol. Isolates were grown aerobically on Nutrient Agar [Biolab, Budapest, Hungary] and incubated overnight at 30°C.

**Table 5:** *Ralstonia *Strains used in this work

Strain	Source
*R. pickettii* JCM5969, NCTC11149, DSM6297, CIP73.23 CCUG3318	Culture Collections

*R. pickettii* CCM2846 CCUG18841	Culture Collections

*R.insidiosa* ATCC4199	Culture Collection

*R.insidiosa* LMG21421	Culture Collection

*R. pickettii* ULC193, ULC194, ULC277, ULC297, ULC298, ULC224, ULC421	Microbiology laboratory of Limerick Regional Hospital (Cystic Fibrosis Patients)

*R. pickettii* ULI785, ULI788, ULI790, ULI791, ULI796, ULI798, ULI800, ULI801, ULI804, ULI806, ULI807, ULI818, ULI159, ULI162, ULI165, ULI167, ULI169, ULI171, ULI174, ULI181, ULI187, ULI188, ULI193	Isolated from various Millipore Purified water systems (Ireland)

*R.insidiosa* ULI821, ULI797, ULI785, ULI181, ULI794, ULI185, ULI166, ULI819, ULI784, ULI163, ULI795	Isolated from various Millipore Purified water systems (Ireland)

*R. pickettii* ULM001, ULM002, ULM003, ULM004, ULM005, ULM006	Isolated from various Millipore Purified water systems (France)

*R. pickettii* ULM007, ULM008, ULM009, ULM010, ULM011	Isolated from various Millipore Purified water systems (Ireland)

*R.insidiosa* ULM008, ULM009	Isolated from various Millipore Purified water systems (Ireland)

### Molecular analysis of genes of Tn*4371*-like ICEs

PCR primers were designed based on the conserved aligned scaffold common to all ICEs characterised in this study and from the consensus sequence of the *Ralstonia pickettii *12J Tn*4371 *ICE using the Primer 3 program [[67], http://frodo.wi.mit.edu/]. All primers are listed in Table 5. The cycling conditions were as follows: initial denaturation (98°C, 2 min); 35 cycles consisting of denaturation [98°C for 15 s], primer annealing [T_A _[estimated primer annealing temperature], 1 min], and extension [72°C, 1 min/kb]; followed by a final extension step [72°C, 10 min]. Amplification was carried out with a GC buffer [in a total reaction of 100 μL containing 0.2 mM deoxynucleoside triphosphates, 100 pmol of each primer, 8 μL of genomic template DNA, and 3 units of Phusion polymerase [New England Biolabs, UK]. Amplification was carried out using a GeneAmp 2400 Thermocycler. Bacterial DNA for PCR amplification was extracted according to Ausubel *et al*. [68]. Amplicons to be sequenced were directly purified from the PCR reaction by the NucleoSpin Extract II kit [Macherey-Nagel, Düren] according to the manufacturer's instructions. Sequence analysis was performed by Euorfins-MWG [Germany] using both the forward and reverse primers listed in Table 3.

### Bioinformatic Analysis of the Tn*4371*-like ICEs in genomes

All analysed DNA sequences were retrieved from the GenBank database http://www.ncbi.nlm.nih.gov.

DNA and protein sequences similar to Tn*4371 *[[13], AJ536756] were detected within the NCBI nonredundant nucleotide and protein databases http://www.ncbi.nlm.nih.gov via blastp and blastn analysis using the original Tn*4371 *sequence as a probe [69]. Assembly and comparison with other Tn*4371*-like sequences was performed with the Artemis Comparison Tool [ACT] [[70], http://www.sanger.ac.uk/Software/ACT]. The complete DNA sequences were also manually annotated to verify the deposited sequence. The similarity of proteins encoded by the element was determined as % aa identities over the entire protein to its Tn*4371 *equivalent via blastp. Unknown ORFs were analysed using InterProScan http://www.ebi.ac.uk/InterProScan/, [71]] to locate motifs or domains where similarity with known proteins was low or absent. Size and total % GC content was determined using the GC-Profile program [[72], http://tubic.tju.edu.cn/GC-Profile/]. Phylogenetic and molecular evolutionary analyses were conducted using genetic-distance-based neighbour-joining algorithms within MEGA version 4.0 [[73], http://www.megasoftware.net/]

### Nucleotide sequence accession numbers

The DNA sequences described in this article have been assigned the accession numbers listed in Table 3.

## Authors' contributions

MRP was responsible for conception of the study, experimental design, data collection, and analysis and preparation of the manuscript. JTP and CCA participated in experimental design, data analysis and preparation of the manuscript. All authors read and approved the final manuscript.

## Supplementary Material

Additional file 1 **Alignment of the conserved domains among the site-specific recombinases of the tyrosine integrase family**. Alignment of the conserved domains among the site-specific recombinases of the tyrosine integrase family from phages, conjugative transposons, plasmids and other sources. R (Arginine) being in Domain I and H (Histidine)-R-Y (Tyrosine) in Domain II. Click here for file

Additional file 2 **Phylogenetic tree of the Integrase proteins from Tn*4371*-like integrases available on the GenBank database and other Phage and ICE integrases**. Phylogenetic tree of the Integrase proteins from available Tn*4371*-like integrases available on the GenBank database and other Phage and ICE integrases. Cluster analysis was based upon the neighbour joining method. Numbers at branch-points are percentages of 1000 bootstrap resamplings that support the topology of the tree. The scale bar represents 0.2 substitutions per nucleotide position. Click here for file

Additional file 3 **Gene numbers for genes in the elements discovered in this study**. The gene numbering for genes of the elements discovered in this study. Genes with yellow background are the scaffold genes of the element. Click here for file

Additional file 4 **Alignment of the first/last 200 bp of Tn*4371*-like ICEs using ClustalW**. **Fig S1a: **Alignment of the first 200 bp of Tn*4371*-like ICEs using ClustalW. **Fig S1b: **Alignment of the last 200 bp of I Tn*4371*-like ICEs using ClustalW. Click here for file
