# Supplementary material for: Novel Tn4371-ICE like element in Ralstonia pickettii and Genome mining for comparative elements
Source: BMC Microbiol. 2009 Nov 26;9:242. doi: 10.1186/1471-2180-9-242 (PMC2789088; doi:10.1186/1471-2180-9-242)
Supplement: Additional file 1 — Alignment of the conserved domains among the site-specific recombinases of the tyrosine integrase family. Alignment of the conserved domains among the site-specific recombinases of the tyrosine integrase family from phages, conjugative transposons, plasmids and other sources. R (Arginine) being in Domain I and H (Histidine)-R-Y (Tyrosine) in Domain II. [file 1471-2180-9-242-S1.PDF]

```
R.pic12J : EAEGLENYVIPLAIRLQFEFAGRRSEIVTL
xerC     : RDRAMLEVMYGAGLRLSELVGLDIKHLDLE
xerD     : RDKAMLEVLYATGLRVSELVGLTMSDISLR
Lambda   : WLRLAMELAVVTGQRVGDLCEMKWSDIVDG
CRE      : ARIRVKDISRTDGGRMLIHIGRTKTLVSTA
P4       : MTRIAVKLSLLTFVRSSELRFARWDEFDFD
HK022    : WLRLAMDLAVVTGQRVGDLCRMKWSDINDN
Tn916    : KNYDEILILLKTGLRISEFGGLTLPDLDFE
P22      : PLKSVVEFALSTGLRRSNIINLEWQQIDMQ
HP1      : RETELAFLYERDIYRLLAECDNSRNPDLG-
pSE211   : ATYHKTEPCKAACKRHTRACPPPCPPACTE
CTn341   : LMEGKLKNAKQELYRDLYLFCAFTGLSFAD
R391     : KNRIYIKLLLLLGGRKGELIQAEKHHFDLQ
                         R
```

Domain I

```
R.pic12J : HVGTHGIRH-RSATDIANSGIPVKVGMALTAHKTVAMFMRYVHTEDD
xerC     : HVHPHKLRH-SFATHMLESSGDLRGVQELLGHANLSTTQIYTHLDFQ
xerD     : KLSPHVLRH-AFATHLLNHGADLRVVQMLLGHSDLSTTQIYTHVATE
Lambda   : PPTFHELRSLSAR-LYEKQ-ISDKFAQHLLGHKSDTMASQYRDDRGR
CRE      : AWSGHSARV-GAARDMARAGVSIPEIMQAGGWTNVNIVMNYIRNLDS
P4       : EVCGHGFRTMARGALGESGLWSDDAIERQLSHSERNVRAAYIHTSEH
HK022    : PPTFHELRSLSAR-LYRNQ-IGDKFAQRLLGHKSDSMAARYRDSRGR
Tn916    : HITPHSLRH-TFCTNYANAGMNPKALQYIMGHANIAMTLNYYAHATF
P22      : DFRFHDLRH-TWASWLVQAGVPISVLQEMGGWESIEMVRRYAHLAPN
HP1      : GQLTHVLRH-TFASHFMMNGGNILVLKEILGHSTIEMTMRYAHFAPS
pSE211   : EARLHDARH-TAATVLLVLGVPDRVVMELMGWSSVTMKQRYMHVIDS
CTn341   : HITWHQSRHTAATTIFLSNGVPIETVSSMLGHKSIKTTQIYAKITKE
R391     : HWSMHDLRR--TMRTRMSAITTQEVAELMIGHSKKGLDAIYNQYQYL
               H  R                                Y
```

Domain II

Additional file 1: Alignment of the conserved domains among the site-specific recombinases of the tyrosine integrase family from phages, conjugative transposons, plasmids and other sources. R (Arginine) being in Domain I and H (Histidine)-R-Y(Tyrosine) in Domain II.
