# Supplementary material for: Novel Tn4371-ICE like element in Ralstonia pickettii and Genome mining for comparative elements
Source: BMC Microbiol. 2009 Nov 26;9:242. doi: 10.1186/1471-2180-9-242 (PMC2789088; doi:10.1186/1471-2180-9-242)
Supplement: Additional file 2 — Phylogenetic tree of the Integrase proteins from Tn4371-like integrases available on the GenBank database and other Phage and ICE integrases. Phylogenetic tree of the Integrase proteins from available Tn4371-like integrases available on the GenBank database and other Phage and ICE integrases. Cluster analysis was based upon the neighbour joining method. Numbers at branch-points are percentages of 1000 bootstrap resamplings that support the topology of the tree. The scale bar represents 0.2 substitutions per nucleotide position. [file 1471-2180-9-242-S2.PDF]

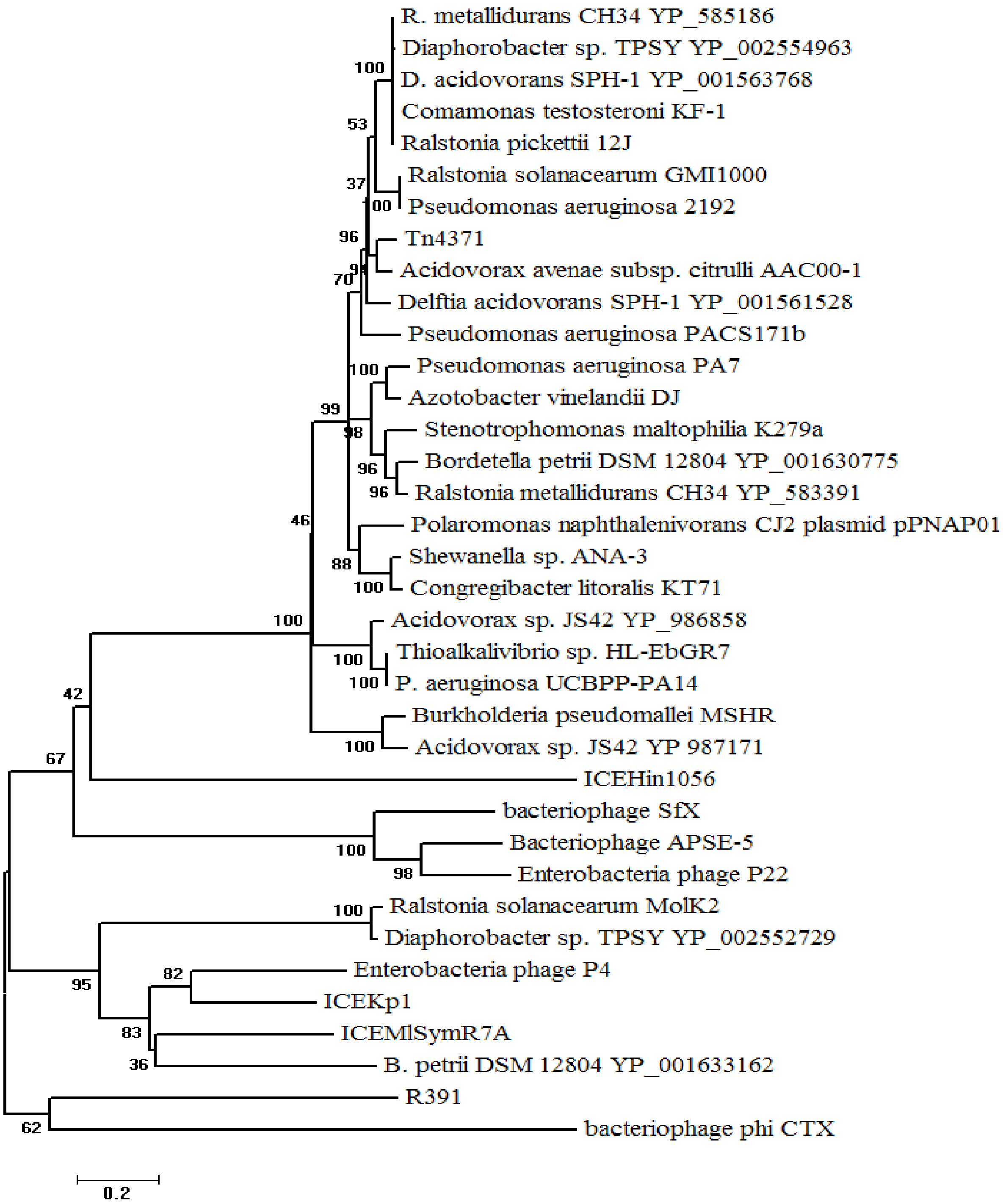

Additional file 2: Phylogenetic tree of the Integrase proteins from available Tn*4371*-like integrases available on the GenBank database and other Phage and ICE integrases. Cluster analysis was based upon the neighbour-joining method. Numbers at branch-points are percentages of 1000 bootstrap resamplings that support the topology of the tree. The scale bar represents 0.2 substitutions per nucleotide position.
