# Supplementary material for: Novel Tn4371-ICE like element in Ralstonia pickettii and Genome mining for comparative elements
Source: BMC Microbiol. 2009 Nov 26;9:242. doi: 10.1186/1471-2180-9-242 (PMC2789088; doi:10.1186/1471-2180-9-242)
Supplement: Additional file 4 — Alignment of the first/last 200 bp of Tn4371-like ICEs using ClustalW. Fig S1a: Alignment of the first 200 bp of Tn4371-like ICEs using ClustalW. Fig S1b: Alignment of the last 200 bp of I Tn4371-like ICEs using ClustalW. [file 1471-2180-9-242-S4.PDF]

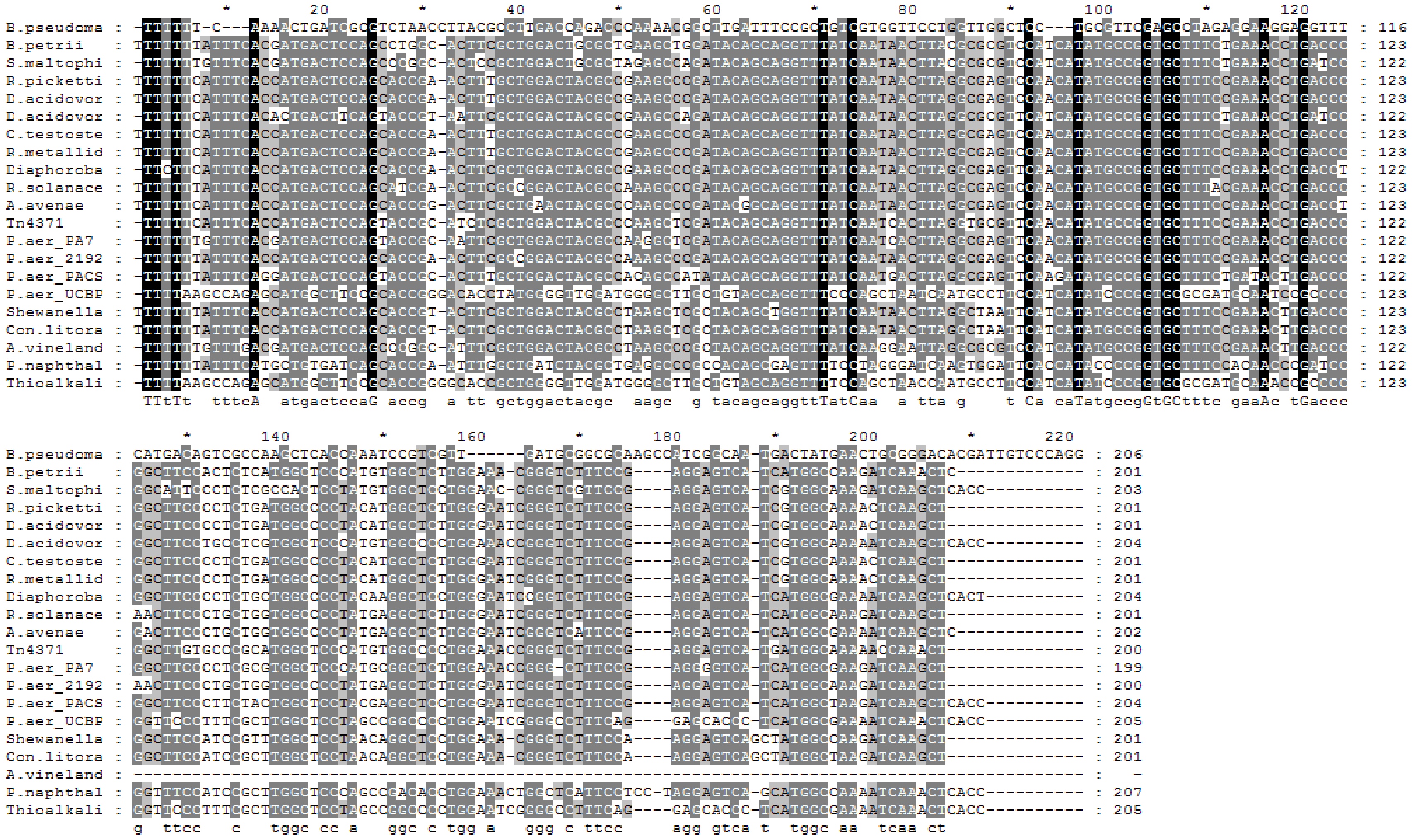

Additional file 4a: Alignment of the first 200bp of ICE$_{Tn\textit{4371}}$*6403* and related Tn*4371*-like ICEs using ClustalW.

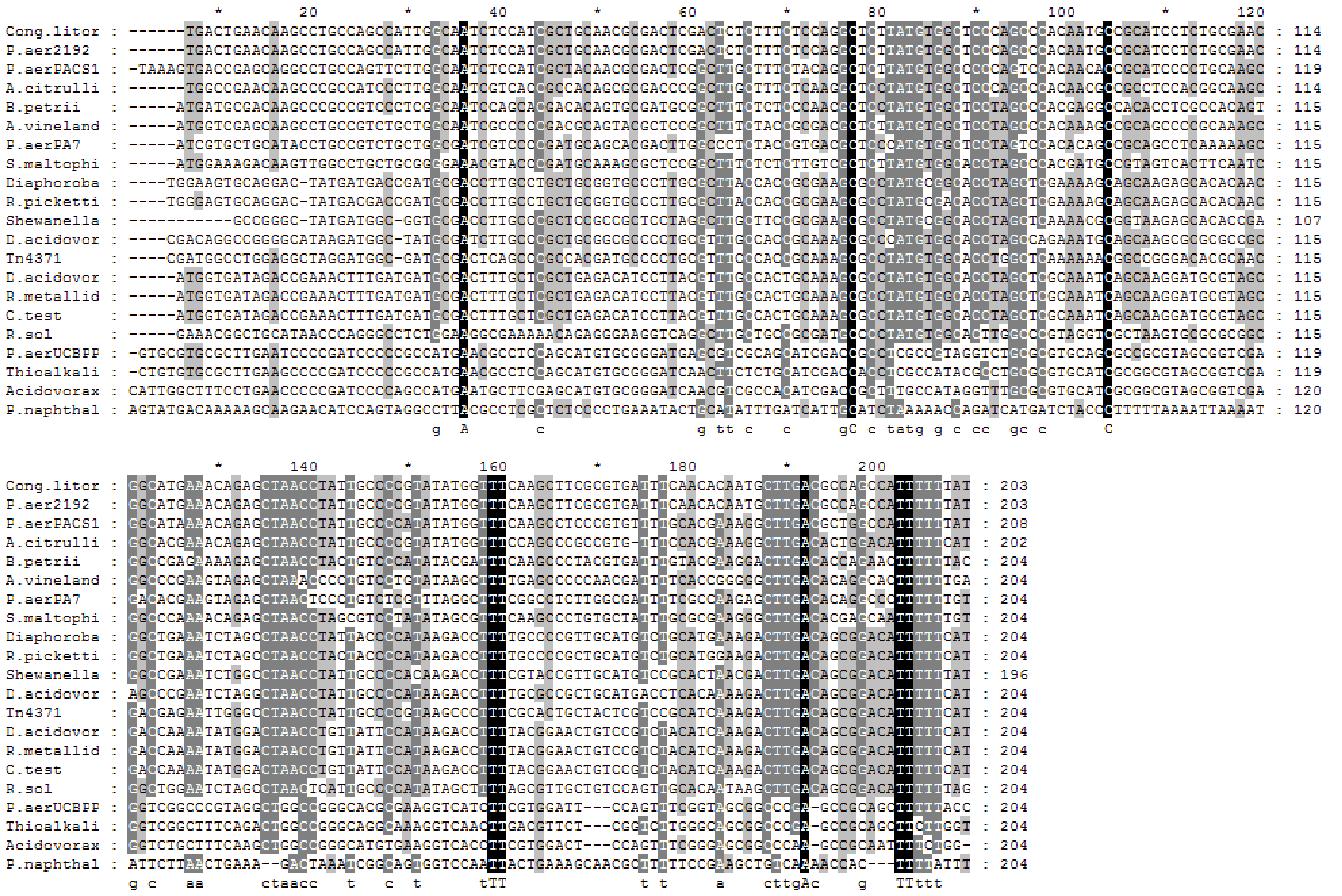

Additional file 4b: Alignment of the last 200bp of ICE$_{Tn\textit{4371}}$*6403* and related Tn*4371*-like ICEs using ClustalW.
